# Development of Flow Cytometry-Fluorescent In Situ Hybridization (Flow-FISH) Method for Detection of PML/RARa Chromosomal Translocation in Acute Promyelocytic Leukemia Cell Line

**Published:** 2017

**Authors:** Fatemeh Zahedipour, Reza Ranjbaran, Abbas Behzad Behbahani, Khalil Tavakol Afshari, Mohammad Ali Okhovat, Gholamhossein Tamadon, Sedigheh Sharifzadeh

**Affiliations:** 1. Diagnostic Laboratory Sciences and Technology Research Center, Faculty of Paramedical Sciences, Shiraz University of Medical Sciences, Shiraz, Iran; 2. Student Research Committee, Shiraz University of Medical Sciences, Shiraz, Iran; 3. Buali Research Institute, Immunology Research Center, Immunogenetic and Cell Culture Lab, Mashhad University of Medical Science, Mashhad, Iran

**Keywords:** Acute promyelocytic leukemia, Flow cytometry, Fluorescent in situ hybridization, PML-RARalpha

## Abstract

**Background:**

Acute Promyelocytic Leukemia (APL) is a subclass of acute myeloid leukemia. The chromosomal aberration in 95% of APL cases is t(15; 17) (q22; q21), which prevents cell differentiation. Characterization of the underlying molecular lesion is valuable in determining optimal treatment strategy. The goal of this study was to develop a new and powerful Flow- FISH technique to detect the long isoform (L) of PML-RARa fusion transcript in NB4 cell line.

**Methods:**

To achieve the best condition for fixation, two different fixatives including 2% paraformaldehyde and 75% ethanol were used. 0.2% Triton X-100 and 0.2% saponin were used for the permeabilization step .In hybridization, a wide range of times and temperatures were used and probe was designed in FRET system. Results were confirmed by fluorescent microscope assay and reverse transcription PCR.

**Results:**

In the present study, a novel technique was successfully optimized that combines in situ hybridization with flow cytometry to detect the presence of PML-RARa transcript. Using standard fixation and permeabilization protocol of 2% PFA and 0.2% saponin gave the best fluorescent results in flow cytometry. Also, results indicated that the optimum time and temperature for hybridization was 2 *hr* at 42°*C*. The results of reverse transcription PCR and fluorescent microscopy confirmed the presence of PML-RARa transcript.

**Conclusion:**

The concordance between the results of Flow-FISH and those of two other techniques including reverse transcription PCR and FISH indicated that this method would be applicable as a diagnostic test for APL in clinical samples and MRD monitoring.

## Introduction

Acute Promyelocytic Leukemia (APL) is a subclass of acute myeloid leukemia. According to the World Health Organization (WHO) classification system, the chromosomal aberration in 95% of APL cases is t(15; 17)(q22;q21). The fusion gene product can recruit various nuclear co-repressors, and block the transcription of genes, which is fundamental to the differentiation process ^1^. The type of the RARa-fusion partner is an essential determinant of response to ATRA, indicating the importance of molecular characterization of APL patients in determining the most appropriate treatment approach and additionally specifying targets for Minimal Residual Disease (MRD) monitoring ^2^.

Molecular methods used for diagnosis of APL include cytogenetic, FISH, immunophenotyping ^3^ and RT-PCR ^4^. In spite of the advantages of cytogenetics, FISH and immunophenotyping using flow cytometry, limited sensitivity for diagnosis and MRD monitoring of APL was observed ^5^. Despite a large number of publications, there has been no unified APL phenotypic profile across the spectrum. As shown in several studies, HLA-DR, CD34 and CD117 expression is variable in APL patients and reliably identifying all APL cases by an FCM screening panel remains a challenge ^6–9^.

G-banding of cells in metaphase permits the detection of translocation but the method is labor-intensive and requires mitotic cells. In comparison to cytogenetics, FISH can be performed on larger numbers of cells. FISH has proven to be more sensitive than G-banding for the diagnosis of some chromosomal abnormalities. FISH limitations include subjective microscopic analysis of fluorescent signals in cell and the low number of cells which can be routinely evaluated (∼ 200 cells) ^10^.

RT-PCR is the most sensitive method with the ability to detect one leukemic cell among 10^4^–10^5^ normal cells. But this method depends on cell lysis and mRNA extraction; therefore, it is not possible to study the cells individually ^11^.

To overcome these technical limitations, the goal of this study was to develop a new and powerful Flow-FISH technique that combines fluorescence in situ hybridization with flow cytometry. This method is relatively fast and cost benefit with high sensitivity. In addition, combining the flow cytometry with FISH gives the chance to examine thousands of cells in a sample.

## Materials and Methods

NB4 was used as a human myeloid leukemic cell line bearing the chromosomal translocation t(15;17) (q22;q21) and HL-60 as a negative control cell line, that retains a promyelocytic morphology but does not express t(15;17). The cells were cultured in culture flasks with RPMI-1640 supplemented with 10% fetal bovine serum (Sigma, USA). Cells were grown at 37°*C* with 5% CO_2_.

### Probe and primer design

Specific primers and FRET probes were designed for PML-RARa fusion mRNA with the following specifications: a forward primer (5′-GGAAGGAGGCAAG GTTGG-3′), a reverse primer (5′-CTGACAGACAAA GCAAGGC-3′) and a pair of FRET probes (5′-FITCTGCTCTGGGTCTCAATGGCTGCCTC-3′; 5′-GGA GGGCTGGGCACTATCTCTTCAGAA-TAMRA-3′).

To evaluate the permeabilization and hybridization methods, a25-base-longs 5′-Fluorescein isothiocyanate (FITC)-labeled probe was used to detect 18S ribosomal RNAs. The sequence was: 5′-TCACCTCTAGCGGCG CAATAC GAAT-3′.

### RNA extraction and cDNA synthesis

The total RNA of NB4 and HL-60 cells was extracted. Subsequently, cDNA synthesis was performed (Bioneer, South Korea) by adding 1 *μg* of RNA to each lyophilized tube which was ready to use.

### qRT- PCR

To confirm the presence of PML-RARa L-form fusion transcript, qRT- PCR was performed. The final volume of the PCR reaction was 25 *μl* and included 5 *μl* cDNA, primers 300 *nmol/L* each and Taq DNA polymerase 2X master mix (Ampliqon, Denmark) 12.5 *μl*. The PCR program was incubation at 95°*C* for 10 *min*, followed by 40 cycles, 95°*C* for 15 *s*, 60°*C* for 30 *s* and 72°*C* for 30 *s*.

### Fixation and permeabilization protocols

In order to determine the best fixation and permeabilization method, two different fixative reagents including 2% paraformaldehyde and 75% ethanol and two different detergents including saponin and Triton X-100 were used. Cells (1×10^6^ cells) were fixed in 500 *μl* of 2% cold and freshly prepared paraformaldehyde in Phosphate Buffered Saline (PBS). Samples were then incubated at room temperature for 15 *min* with gentle shaking. Then, samples were washed in PBS and centrifuged. For cell permeabilization, 200 *μl* of saponin (Sigma, USA) at concentration of 0.2% was added to each tube and incubated for 10 *min* at 4°*C*.

In a separate experiment, cells were fixed in 500 *μl* of 75% ethanol and incubated at 4°*C* for 15 *min*. Cells were then permeabilized by adding 200 *μl* of 0.2% Triton X-100 and incubated for 5 *min* at room temperature. The samples were then washed with PBS.

### In situ hybridization

The cells were suspended in 50 *μl* of hybridization buffer containing 20XSSC, 50% formamide, 50X Denhardt’s solution, 2 *M* NaH_2_PO_4_/Na_2_HPO_4_ and, 10% Dextran sulfate. 0.3 *μg/ml* of heated and denatured donor and acceptor probes were added to the cells. Different incubation times (1, 2 and 20 *hr*) and temperatures (25, 37, 42 and 44°*C*) were studied. The cells were then pelleted and washed with 2XSSC and 0.1XSSC for 10 *min* each to remove non-specific and unbound probes. Finally, the cells were resuspended in 1 *ml* 1XPBS buffer for flow cytometric analysis.

### Flow cytometric analysis

Samples were analyzed on a FACS Calibur flow cytometer (Becton Dickinson, USA). FITC and TAMRA were detected using 530/30 and 585/42 *nm* filters. For each sample, 10000 events were acquired and the data were analyzed using Cell Quest software (BD, USA). Data were expressed as the percentage of positive cells bearing PML-RARa fusion transcript and measured in FL2 channel (TAMRA) of flow cytometer and also positive cells for 18S rRNA in FL1 channel (FITC) and geometric mean fluorescence intensity.

### Fluorescent microscopy

Following hybridization, the cells were washed with 0.1 x SSC and dispensed into two separate tubes to perform flow cytometry and microscopic examination, respectively. Cells were mounted on glass slide and evaluated on fluorescent microscope (Zeiss, Germany) with appropriate filter sets.

## Results

### Flow-FISH

Using standard fixation and permeabilization protocol of 2% PFA and 0.2% saponin gave the best fluorescent results compared with other groups.

**Figure 1.**
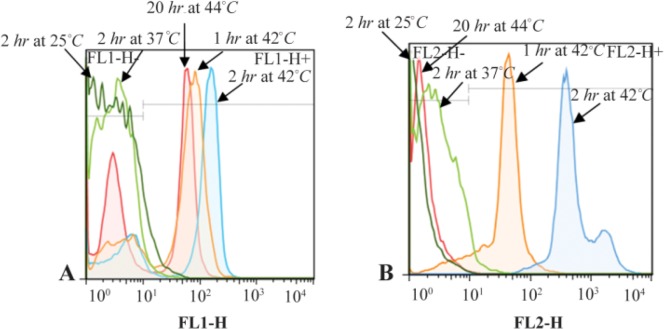
Flow cytometric histograms to compare the effect of different hybridization times and temperatures on fluorescence intensity. A. Flow cytometric histograms of 18S rRNA probe. B. Flow cytometric histograms of PML-RARa FRET probe.

**Figure 2.**
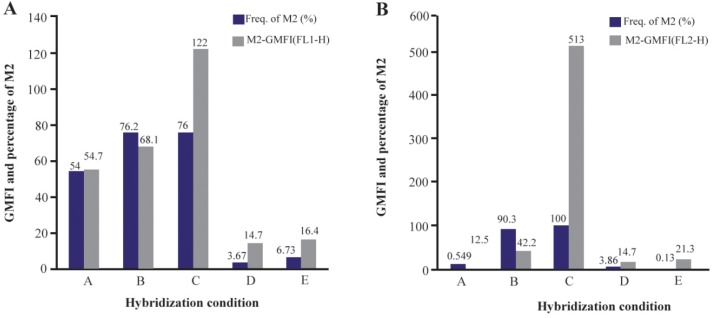
Flow cytometric analysis of the effect of different hybridization times and temperatures on GMFI and fluorescence intensity with 18S rRNA probe (I) and PML-RARa probe (II). A. Hybridization at: A) 44°*C* for 20 *hr*; B) 42°*C* for 1 *hr*; C) 42°*C* for 2 *hr*; D) 37°*C* for 2 *hr*; E) 25°*C* for 2 *hr*.

**Figure 3.**
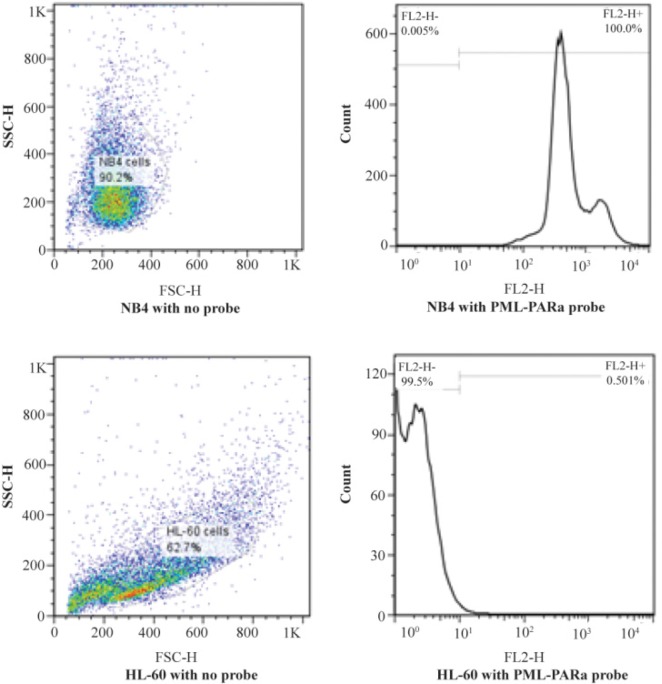
Flow cytometric histograms of NB4 cells (A) and HL-60 cells (B). Hybridization with PML-RARa probe at 42°*C* for 2 *hr.*

The most promising results with 18S rRNA probe that gave the highest GMFI and fluorescence intensity were obtained at 42°*C* (M1=24.8%, M2=75.2%) and 44°*C* (M1=23.8%, M2=76.2%) and with PML-RARa probe, the best result was obtained only at 42°*C*. At first, the cells were incubated at 42°*C* for 1 *hr* (M1=9.74%, M2=90.3%) (Figures 1 and 2). In another test, incubation time was raised to 2 *hr* where the GMFI and percentage of M2 increased dramatically (M1=0.005%, M2=100%) (Figure 3).

### Fluorescent microscopy

**Figure 4.**
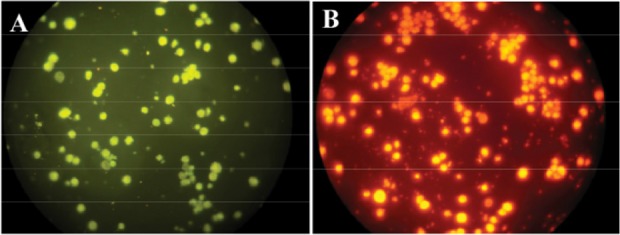
FISH test. NB4 cells with PML-RARa FRET probes in green (A) and red channel (B).

Flow FISH results were verified by fluorescent microscope. Following hybridization, examination of cells by fluorescent microscopy using appropriate filters showed the fluorescence signal of TAMRA labeled probe (red fluorescent) and FITC labeled probe (green fluorescent) as shown in figure 4.

### qRT-PCR

**Figure 5.**
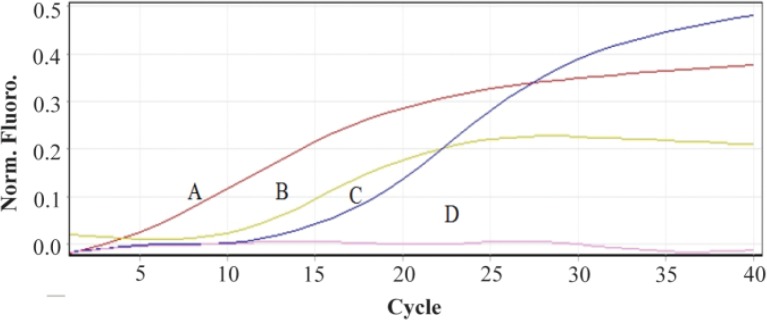
Real time PCR in order to confirm the presence of PMLRARa mRNA (L-isoform). A) Neat PML-RARa mRNA extracted from NB4 cells. B, C) PML-RARa mRNA in ½ and 1/10 dilutions. D. mRNA extracted from HL-60.

The qRT-PCR technique confirmed the presence of PML-RARa L-form fusion transcript in NB4 cell by means of specific primers designed around FRET probe binding site. Also, by comparing the results of Flow-FISH with qRT-PCR, Flow-FISH was found to be 100% specific (Figure 5).

## Discussion

As the APL is a highly aggressive disease, its accurate diagnosis and treatment is of critical importance. Also, direct targeted therapy with ATRA has transformed the management of APL over the last decades ^12^. Several methods are available for diagnosis of APL, each with distinctive advantages and disadvantages.

In cytogenetic and FISH analysis, direct chromosomal examination is performed. Therefore, falsely positive or negative results may be obtained. For example, in some cases where APL was shown to have a normal karyotype, nested RT-PCR was found to detect both PML-RARa and reciprocal RARa-PML fusion transcripts, implying that the t(15;17) was present, but cytogenetic analysis was not able to detect this translocation ^13^. Also, in some other cases, residual normal marrow elements whose growth is similar to APL blasts can lead to a falsely normal karyotype ^14^. A potential advantage of the FISH technique is that it permits the evaluation of both interphase and metaphase cells. However, as the final examination is performed by fluorescent microscope, the numbers of cells which are examined are limited.

In order to facilitate rapid diagnosis of APL, flow cytometry has been extensively used and studied. However, studies revealed that the most widely cited immunophenotypic features of APL are not found in all APL cases and are insufficient for separating APL from other types of Acute Myelocytic Leukemia (AML) ^15^.

Among the various molecular techniques available, RT-PCR provides relatively sensitive and rapid confirmation of clinical diagnosis of APL. However, since the cells are lysed, it is not possible to associate a signal with an individual cell or determine the exact number of leukemic cells in bone marrow which is important in MRD monitoring of APL ^16^.

In Flow-FISH, each cell is independently examined, thereby enabling the detection of cells containing specific chromosomal aberration and the quantification of the number of leukemic cells in a population. In addition, flow cytometry permits the analysis of a large number of cells and also the detection of rare cells in mixed solutions. However, the Flow-FISH technique is relatively new and like most new techniques, various modifications to the original protocols are needed to be made ^17,18^.

## Conclusion

In the present study, for the first time, a new Flow-FISH technique was developed to detect PML/RARa fusion transcript in NB4 cell line. The concordance between the results of Flow-FISH and those of two other techniques including reverse transcription PCR and FISH and also its high specificity indicates that this method can be applicable as a diagnostic test for APL in clinical samples and MRD monitoring.
